# Integrating Fine-Tuning and Retrieval-Augmented Generation for Healthcare AI Systems: A Scoping Review

**DOI:** 10.3390/bioengineering13020225

**Published:** 2026-02-14

**Authors:** Bernardo G. Collaco, Prabha Srinivasagam, Cesar A. Gomez-Cabello, Syed Ali Haider, Ariana Genovese, Nadia G. Wood, Sanjay Bagaria, Mark A. Lifson, Antonio Jorge Forte

**Affiliations:** 1 Division of Plastic Surgery, Mayo Clinic, 4500 San Pablo Rd S, Jacksonville, FL 32224, USA; collaco.bernardo@mayo.edu (B.G.C.);; 2 Department of Radiology AI IT, Mayo Clinic, Rochester, MN 55905, USA; 3 Department of Surgery, Mayo Clinic, Jacksonville, FL 32224, USA; 4 Center for Digital Health, Mayo Clinic, Rochester, MN 55905, USA; 5 Department of Artificial Intelligence and Informatics, Mayo Clinic, Jacksonville, FL 32224, USA

**Keywords:** Artificial Intelligence, large language models, retrieval-augmented generation, fine-tuning, parameter-efficient fine-tuning, healthcare

## Abstract

(1) Background: Large language models (LLMs) show promise in healthcare but are constrained by hallucinations, static knowledge, and limited domain specificity. Fine-tuning (FT) and retrieval-augmented generation (RAG) offer complementary solutions, with FT embedding domain reasoning and RAG enabling dynamic, up-to-date knowledge access. Hybrid FT + RAG frameworks have been proposed to improve factual accuracy and clinical reliability. This scoping review synthesizes current evidence on such hybrids in healthcare AI. (2) Methods: The search across PubMed, IEEE Xplore, Google Scholar, and Embase identified studies implementing explicit FT + RAG hybrids in healthcare or biomedical tasks. Eligible studies reported empirical evaluations of LLM performance or behavior. Data were extracted on base models, FT strategies, RAG architectures, applications, and performance outcomes. (3) Results: Seven studies met inclusion criteria. FT + RAG systems consistently outperformed FT-only or RAG-only approaches across QA, clinical summarization, report generation, and decision support tasks. Parameter-efficient FT methods (e.g., LoRA) were common, while RAG implementations varied (dense, hybrid, hierarchical, multimodal, federated). Reported benefits included improved accuracy, reduced hallucination, and greater clinician preference and feasibility in protected settings. (4) Conclusions: FT + RAG frameworks represent a promising direction for clinically grounded healthcare AI, combining domain-specific reasoning with transparent, up-to-date retrieval. Future work should prioritize standardized evaluation, workflow integration, and governance to enable safe deployment.

## 1. Introduction

The rapid advancement of Large Language Models (LLMs) has introduced a transformative paradigm shift across numerous sectors, with significantly impact on healthcare [1,2]. LLMs are capable of processing vast amounts of unstructured clinical text, generating human-like responses, and supporting complex tasks such as medical documentation, diagnostics, and patient education [3]. This capability promises to enhance workflow efficiency and improve patient care by providing clinicians with sophisticated decision-support tools [1].

Despite their general-purpose proficiency, the direct application of LLMs in high-stakes clinical environments presents significant challenges. General models often lack the deep domain expertise required for specialized medical tasks and are prone to generating hallucinations (factually incorrect or non-existent information), which poses a critical risk to patient safety and clinical trust [4,5]. Furthermore, the knowledge embedded within these models is static, quickly becoming outdated in the face of rapidly evolving medical literature and clinical guidelines [6]. To bridge this gap between general capability and clinical necessity, researchers have focused on knowledge adaptation strategies to ground LLMs in the medical domain.

Two primary strategies have emerged for adapting LLMs to specialized medical contexts: Fine-Tuning (FT) and Retrieval-Augmented Generation (RAG). FT involves further training a pre-trained model on domain-specific datasets, allowing it to learn specialized patterns, terminology, and reasoning capabilities [7]. While effective for deeply embedding domain knowledge, FT is computationally expensive, risks the catastrophic forgetting of general knowledge, and results in a model whose knowledge remains static until the next retraining cycle [8]. Conversely, RAG dynamically connects the LLM to external, up-to-date knowledge bases, enabling it to retrieve relevant information and use it to inform its generated response [9]. RAG offers greater transparency and information currency but may lack the deep, specialized reasoning acquired through FT [10,11].

**Figure 1:**
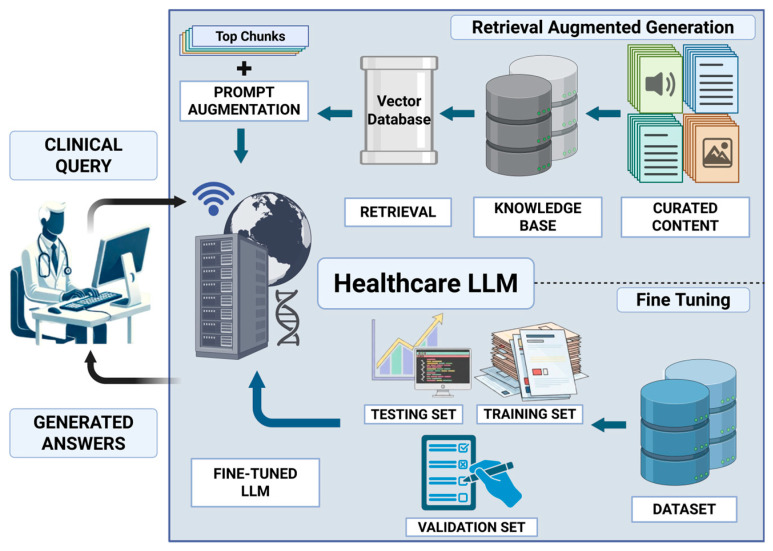
Hybrid RAG and FT architecture for healthcare LLMs. Clinical queries are processed by a fine-tuned healthcare LLM (bottom panel) that has been adapted using supervised domain-specific training data (training, validation, and testing sets). In parallel, a retrieval pipeline (top panel) queries a curated healthcare knowledge base indexed in a vector database to identify the top-K most relevant document chunks. These retrieved passages are incorporated into the input through prompt augmentation, enabling dynamic grounding in up-to-date external evidence. The fine-tuned LLM then generates responses by jointly leveraging its domain-adapted internal representations and the retrieved contextual knowledge. Created in BioRender. Collaco, B. (2025) https://BioRender.com/ecvmjvt.

The limitations inherent in both standalone FT and RAG approaches have driven the development of hybrid frameworks that strategically combine the strengths of both [12]. These hybrid systems aim to leverage the deep domain adaptation and reasoning capabilities provided by FT with the factual grounding, transparency, and real-time knowledge access afforded by RAG [13]. By integrating these strategies, hybrid models offer a promising pathway toward creating AI systems that are not only accurate and reliable but also flexible and safe for deployment in dynamic clinical settings (Figure 1).

While FT-only and RAG-only frameworks have each been extensively studied and reviewed in isolation, substantially less attention has been devoted to systems that explicitly integrate both strategies within a unified clinical pipeline. This scoping review aims to characterize and synthesize the current landscape of frameworks that adopt both strategies (hybrid FT + RAG) in healthcare AI. Specifically, we analyze the reported use cases, performance characteristics, and implementation trade-offs. By synthesizing the empirical evidence, this review provides a critical assessment of how these integrated approaches are advancing the factual accuracy, domain grounding, and reliability of LLM outputs in clinical and biomedical tasks, thereby informing future research and guiding the responsible deployment of next-generation AI in medicine.

## 2. Materials and Methods

This scoping review was conducted in accordance with established methodological guidance for scoping reviews, including the Joanna Briggs Institute framework [14], and is reported following the PRISMA-ScR checklist [15]. The study protocol was prospectively registered in the Open Science Framework (OSF) and is available at: https://doi.org/10.17605/OSF.IO/GBEKT.

### 2.1. Eligibility Criteria

Studies were eligible if they examined LLM-based systems applied to healthcare or biomedical contexts, such as clinical care, medical education, biomedical research, or electronic health record (EHR)-related workflows. This review focused on explicitly integrated hybrid FT + RAG systems, defined operationally as systems that met all of the following criteria: (I) Model-level parameter adaptation (FT): the base generative LLM underwent direct parameter updating through full FT or parameter-efficient fine-tuning (PEFT) methods (e.g., LoRA, QLoRA, or related adapter-based approaches); (II) Retrieval-augmented generation (RAG): the system incorporated external knowledge through a retrieval pipeline used at inference time to condition generation (e.g., embedding-based, lexical, or hybrid retrieval over a specified corpus/knowledge source); and (III) Integrated architecture: FT and retrieval were described and evaluated as part of a single end-to-end system (i.e., retrieval was not used solely for dataset construction, and FT was not evaluated as a separate component disconnected from an inference-time retrieval pipeline).

This definitional boundary was selected to map a methodologically distinct and underexplored subclass of healthcare LLM systems that combine domain adaptation via parameter updates with dynamic knowledge grounding via retrieval, and to identify evidence gaps, implementation patterns, and evaluation practices within this subclass.

Eligible studies were required to report empirical evaluation of the integrated FT + RAG system relevant to clinical or biomedical use, including (but not limited to) task performance (e.g., accuracy or F1), hallucination reduction, faithfulness/grounding metrics, safety- or reliability-oriented outcomes, task-specific clinical/biomedical endpoints, or clinician-informed assessments. We included peer-reviewed articles and high-quality preprints that provided sufficient methodological detail to assess eligibility and characterize the FT + RAG workflow. We included healthcare applications such as clinical decision support, documentation and summarization, diagnostics or triage support, patient-facing information, biomedical question answering, and EHR-focused tasks.

Studies were excluded if they did not implement an integrated hybrid FT + RAG architecture, including those relying solely on prompt-based retrieval, retrieval without model-level adaptation, or FT without retrieval. Additional exclusions applied to studies focused on non-healthcare domains, those lacking empirical evaluation of outcomes relevant to clinical utility or safety, and works limited to theoretical discussion, editorials, or commentaries without implementation or validation. Studies centered on non–LLM-based systems, publications with insufficient methodological transparency, inaccessible full text, or redundant reporting of previously published results were also excluded to ensure methodological rigor and relevance.

### 2.2. Study Screening

A structured literature search was conducted across PubMed, IEEE Xplore, Google Scholar, and Embase on 15 December 2025. Database-specific search strings were developed using combinations of terms related to LLMs, FT (including parameter-efficient methods such as PEFT, LoRA, and QLoRA), RAG, and clinical or biomedical applications. The complete search strategy can be found in Supplementary File S1. All records retrieved from the database searches were aggregated and de-duplicated prior to screening using Endnote [16]. Titles and abstracts were first reviewed to exclude clearly irrelevant studies, including those unrelated to healthcare, those not involving LLM-based systems, or those lacking any form of model-level knowledge integration. Full-text review was then performed for all remaining articles to assess eligibility based on predefined inclusion and exclusion criteria. More than 20,000 results were retrieved for Google Scholar, but only the first 100 were chosen for review based on its relevance-based sorting [17]. The screening was conducted by two authors (B.C. and S.P.) and disagreements were resolved through discussion, with a third involved when necessary (CGC).

### 2.3. Data Extraction and Synthesis

For each included study, two independent reviewers (B.C. and S.H.) manually extracted the baseline model, knowledge integration strategies, clinical application domain (e.g., coding, documentation, diagnosis, decision support), reported benefits and limitations, and any quantitative performance changes when available (e.g., accuracy deltas, odds ratios, hallucination-related outcomes). A formal meta-analysis was not performed due to substantial heterogeneity across model architectures, adaptation strategies, datasets, evaluation metrics, and clinical use cases; therefore, a narrative synthesis was used to qualitatively summarize and compare findings.

To ensure interpretability and comparability across studies, FT strategies were categorized according to their optimization paradigm rather than author-defined terminology. Specifically, approaches were classified as PEFT using LoRA or QLoRA, PEFT combined with reinforcement- or preference-based alignment, RAG-aware FT explicitly designed to leverage retrieved contexts, or federated PEFT when FT was performed across decentralized institutions without sharing raw data. Similarly, RAG approaches were categorized according to their retrieval strategy and architectural complexity. RAG implementations were classified as dense, hybrid (sparse + dense), hierarchical, multimodal, adaptive, or federated, depending on the presence of embedding-based retrieval, keyword-based retrieval (e.g., BM25), multi-stage retrieval pipelines, multimodal inputs, dynamic retrieval policies, or decentralized infrastructures.

## 3. Results

### 3.1. Study Screening and Selection

**Figure 2:**
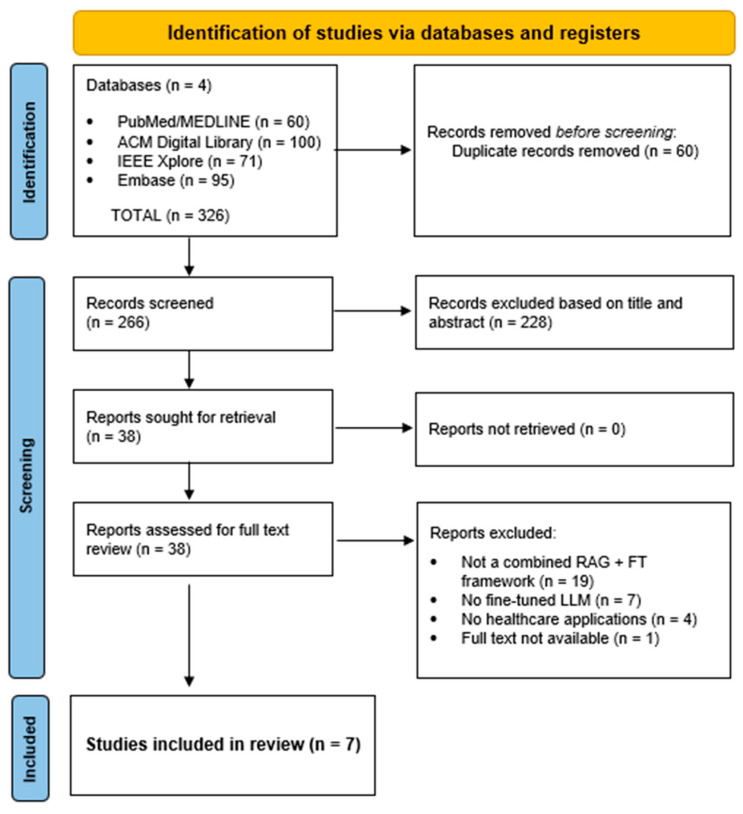
PRISMA Flow Chart of the study selection process.

The initial search across three databases identified 326 records. After duplicate removal, 266 studies underwent title and abstract screening, of which 38 were retained for full-text review. Of these, articles were excluded due to the absence of an explicit combined RAG + FT framework (*n* = 19), lack of parameter adaptation of the generative LLM (*n* = 7), absence of healthcare applications (*n* = 4), or unavailable full text (*n* = 1). Ultimately, seven studies met all inclusion criteria and were included in the final qualitative synthesis on 16 December 2025 [8,18,19,20,21,22,23]. The screening process is summarized in Figure 2.

### 3.2. Baseline Characteristics of Included Studies

Baseline LLMs varied across studies, reflecting differences in experimental scope and comparative objectives. While some works evaluated multiple adaptation strategies beyond RAG + FT [8,18], LLaMA-based architectures were the most commonly used foundation models. Despite methodological heterogeneity, all studies aimed to improve factual accuracy, domain grounding, and reliability of LLM outputs in clinical or biomedical tasks. The included studies covered a range of clinical applications, including medical question answering (QA) [8,18,21,23], clinical summarization [22], automated report generation [20], medical chatbot interactions [18], and multimodal medical reasoning [19]. The baseline characteristics of included studies are presented in Table 1.

Across studies, PEFT approaches, primarily LoRA or QLoRA, were consistently adopted, with several works relying on curated medical QA datasets (e.g., Meadow-MedQA, MedMCQA, and MedQuAD). This reflects a shared emphasis on computational efficiency and practical deployment in resource-constrained healthcare environments [8,18]. Several studies further extended PEFT with complementary optimization strategies, including federated FlexLoRA to enable privacy-preserving training [19], reinforcement-learning–based alignment to reduce unsafe or inconsistent outputs [20], and direct preference optimization (DPO) to better align model generations with clinically preferred and evidence-consistent behaviors [23].

In contrast, substantial variability was observed in RAG implementation strategies, underscoring the importance of categorizing RAG beyond a single umbrella definition. Dense RAG pipelines relied exclusively on embedding-based retrieval from vector databases and were primarily employed in studies focused on medical QA and chatbot performance, drawing on curated textual resources such as medical textbooks, encyclopedias, peer-reviewed journals, and benchmark datasets including USMLE-style MedQA [8,18]. Hybrid RAG architectures (sparse + dense) combined lexical retrieval (e.g., BM25-style matching) with semantic embeddings and were commonly used for tasks requiring structured information extraction or summarization, such as SOAP note generation and biomedical QA [21,22]. Hierarchical and multimodal RAG frameworks, such as the RAG-LLM and MMed-RAG approaches proposed by Kuo et al. (2025) [20] and Xia et al. (2024) [23], represented the most advanced implementations, employing multi-stage retrieval pipelines spanning documents, snippets, and modality-specific indices, and supporting heterogeneous data sources including EHRs, claims data, and medical images. In addition, DF-RAG extended conventional RAG paradigms by leveraging federated knowledge graphs (FKGs) as the retrieval mechanism, enabling decentralized and ontology-aligned retrieval across institutions while preserving data privacy and enhancing interpretability. Retrieved information included structured and semi-structured clinical data, such as EHRs, imaging reports, treatment histories, and diagnostic outcomes, organized using domain-specific ontologies including UMLS and SNOMED-CT [19].

### 3.3. Impact on Model Performance

Across all included studies, FT + RAG approaches consistently outperformed either method used in isolation. In controlled benchmarking settings, FT + RAG pipelines improved answer accuracy, factual consistency, and robustness to unseen medical knowledge, particularly for knowledge-intensive tasks such as medical QA and clinical summarization. Pingua et al. (2025) showed that FT + RAG configurations achieved higher lexical and semantic similarity scores, including improvements in BLEU, ROUGE, BERTScore, and negation-aware semantic similarity, while remaining computationally efficient in low-resource settings [8]. Similarly, the RAG-based medical chatbot proposed by Bora et al. (2024) achieved up to 57% exact-match accuracy on multiple-choice medical QA benchmarks, with Mistral-7B consistently outperforming alternative backbone models [18]. CLINICSUM, developed by Neupane et al. (2024), achieved higher ROUGE and BERTScore metrics than proprietary GPT-based baselines when generating SOAP summaries from patient–doctor conversations and was preferred by clinical experts in 61% of pairwise evaluations, indicating improved factual accuracy and clinical utility [22].

More complex retrieval architectures yielded additional performance gains. Hierarchical pipelines that decomposed retrieval into multiple stages (e.g., document-level followed by snippet-level retrieval) improved performance in biomedical QA benchmarks, as demonstrated by the CPS framework proposed by Gao et al. (2024), which achieved ROUGE-2 F1 = 0.558 and ROUGE-SU4 F1 = 0.573 on ideal answer generation [21]. Multimodal hybrid systems extended these benefits to vision–language tasks, with domain-aware and adaptive retrieval strategies improving factual accuracy in medical visual QA (18.5%) and report generation (69.1%) across radiology, pathology, and ophthalmology datasets [23]. In particular, the multimodal RAG-LLM proposed by Kuo et al. (2025) reduced hallucination rates to approximately 6%, representing a greater than 40% reduction relative to prompt-only baselines, while decreasing the report drafting time by 75%, highlighting the practical efficiency of hybrid systems in real-world settings [20].

Finally, federated frameworks addressed key privacy and generalizability constraints. DF-RAG demonstrated that federated FT combined with FKG-based retrieval can improve diagnostic reliability while enabling cross-institutional collaboration without sharing raw data [19]. Overall, these findings indicate that FT + RAG frameworks offer performance advantages and may be well suited for clinical deployment by combining efficient model adaptation with access to relevant external knowledge. The summary of qualitative and quantitative reports is described in Table 2.

## 4. Discussion

### 4.1. Overview of Model Adaption in Healthcare

Recent literature reveals distinct application domains and performance characteristics for AI models that employ FT or RAG in healthcare, with each approach demonstrating unique advantages for specific clinical use cases.

FT approaches, particularly those leveraging parameter-efficient methods such as PEFT, LoRA, and QLoRA [12,18], are efficient in standardized, task-specific applications that demand deep domain expertise. A prominent example is medical coding automation, where fine-tuned models accurately handle complex clinical documentation by learning highly specialized patterns [24]. These tailored models also perform strongly in clinical documentation processing, medical report generation, and named entity recognition for medical terminology [24,25].

Moreover, FT is essential for aligning LLMs with medical knowledge, enhancing accuracy, safety, and clinical relevance [26]. However, several limitations have emerged. Studies show that FT on publicly available medical datasets may yield limited performance gains and introduce risks such as catastrophic forgetting, where models lose previously acquired general knowledge during FT [27].

To address these constraints, researchers have increasingly explored RAG as an alternative strategy for clinical adaptation. For instance, Dorfner et al. (2025) recommend RAG for its flexibility and effectiveness in dynamic clinical contexts [27]. RAG-only implementations are particularly well-suited to dynamic, knowledge-intensive healthcare applications requiring real-time external data access. These models have outperformed baselines in gastrointestinal imaging diagnosis, emergency department outcome prediction, and clinical decision support [28]. RAG systems also excel in differential diagnosis and medical information retrieval, generating more accurate and contextually relevant responses [29]. RAG further improves workflow efficiency by automating literature reviews and evidence synthesis tasks. It has also been shown to support more equitable and personalized medical content generation by integrating context-specific data for diverse patient populations [30], with promising results in areas like postoperative patient management [31].

A key strength of RAG is its transparency and traceability, allowing clinicians to verify information sources, thereby enhancing trust and diagnostic accuracy [32]. However, a recent review suggests that traceability does not automatically translate to comprehensive safety assurance in practice: most surveyed studies do not evaluate bias, even though bias amplification is a central concern in clinical AI. While RAG is repeatedly associated with hallucination reductions, the review emphasizes that this effect varies by backbone model, and that RAG does not eliminate bias originating from underlying model training data [10].

Quantitative evaluations further support RAG’s advantages. A systematic review and meta-analysis of 20 peer-reviewed studies found that RAG-based systems achieved a statistically significant improvement in performance over baseline LLMs, with an odds ratio of 1.35 (95% CI: 1.19–1.53, *p* = 0.001) [33]. Unlike FT, which is resource-intensive, inflexible, and limited by static training data, RAG retains the original LLM architecture while dynamically incorporating up-to-date knowledge into each query. This results in greater flexibility, lower cost, and real-time responsiveness to clinical updates [34]. Additionally, RAG has proven particularly effective in reducing hallucinations and improving clinical accuracy. For example, it increased Vicuna-7 B’s medical QA performance from 44.46% to 48.54% and achieved 69.68% accuracy in the i-MedRAG system without additional FT [35,36]. RAG also achieved 100% accuracy across 190 million variants in large-scale tasks such as genomic variant annotation, outperforming fine-tuned models that struggled to generalize beyond narrow tasks [37].

Despite these advantages, FT still offers valuable task-specific reasoning capabilities. Therefore, a growing body of research supports hybrid approaches by integrating RAG with fine-tuned components. These systems combine RAG’s contextual precision and scalability with FT’s deep domain adaptation, resulting in generalizable and highly targeted models, particularly valuable in clinical decision support and complex healthcare domains [13,37,38].

Recent work by Soudani et al. (2024) systematically compares FT and RAG as strategies for addressing low-frequency or underrepresented factual knowledge, a significant challenge in specialized fields like medicine [13]. Their results indicate that while FT can benefit smaller models, RAG consistently outperforms FT, especially when domain-specific training data is sparse. They also introduce Stimulus RAG, an enhanced variant incorporating targeted cues from retrieved documents, boosting response quality without retraining the model [13].

Complementary research highlights FT-enhanced RAG architectures that use PEFT techniques such as LoRA and QLoRA to integrate structured retrieval with domain reasoning [12]. Though initially tested in fields like social services, these hybrid designs show great promise in healthcare applications such as real-time patient-facing assistants, low-resource clinical support systems, and health information delivery, where retrieval accuracy, domain specificity, and deployment efficiency are all critical [8,12,13,33].

Building on these developments, recent comparative studies reveal that RAG + FT hybrid approaches consistently outperform either method alone, particularly when applied to specialized medical datasets [18]. Notable gains have been observed across multiple LLMs, including Llama-3.1-8B, Gemma-2-9B, and Mistral-7B [8]. Additionally, Lopez et al. (2025) introduced CLEAR (Clinical Entity Augmented Retrieval), a hybrid model that combines FT with RAG for extracting clinical entities from unstructured EHRs [39]. This study exemplifies an FT strategy outside the base foundation model through additional task-level training [39].

However, while RAG is often discussed as a safer alternative to parameter updates, Amugongo et al. (2025) cautions that data leakage remains possible through both the foundation model (pretraining/FT datasets) and the retrieval dataset, which in clinical settings may include diagnoses, medications, and private patient information [10]. Consistent with this concern, the same review reports that the majority of surveyed healthcare RAG studies do not address privacy issues, with only a small minority implementing explicit privacy mitigations. In addition, ethical and safety considerations remain under-assessed in current healthcare RAG literature. Most papers do not assess bias, despite bias being a central concern for clinical equity; even where RAG reduces biased content relative to an LLM alone in some settings, bias is not eliminated because it is partially attributable to underlying model training data. Similarly, only a small subset of studies explicitly evaluated safety against intentional or unintentional harms, emphasizing the need for broader safety evaluation practices when implementing hybrid RAG and FT approaches [10,40,41].

Together, these advances underscore the growing role of hybrid RAG and FT frameworks in healthcare AI. These approaches enable scalable, cost-effective, and context-aware systems capable of maintaining up-to-date medical knowledge while supporting various tasks, from emergency triage and diagnostic coding to personalized treatment planning and real-time clinical decision-making [8,18,39].

### 4.2. RAG + FT Frameworks in Healthcare AI

Based on the studies included in this review, we can map a preliminary set of recurring design motivations and implementation choices among explicitly integrated hybrid systems that combine model-level FT with inference-time RAG in healthcare and biomedical contexts. Although the evidence base remains limited, the included studies consistently frame hybridization as a pragmatic strategy to reconcile three competing demands: (i) improved factual reliability in high-stakes settings, (ii) domain-specific adaptation without prohibitive computational cost, and (iii) deployment feasibility under privacy, governance, and data-evolution constraints. Together, these motivations support a shift away from purely static adaptation or purely retrieval-driven augmentation toward architectures that deliberately distribute “knowledge” and “adaptation” across modular components.

A central finding of this scoping synthesis is the scarcity of published work that both implements an explicitly integrated FT + RAG pipeline and reports empirical evaluation in clinical or biomedical tasks [8,18,19,20,21,22,23]. The small number of included studies therefore reflects an evidence gap at the intersection of two comparatively mature streams, FT-only and retrieval-only approaches, rather than a lack of conceptual interest in hybrid systems. This gap indicates that integrated FT + RAG remains an emerging methodological direction in healthcare, and it reinforces the need for more empirical validation, comparative evaluation, and real-world testing with clinically relevant endpoints.

**Figure 3:**
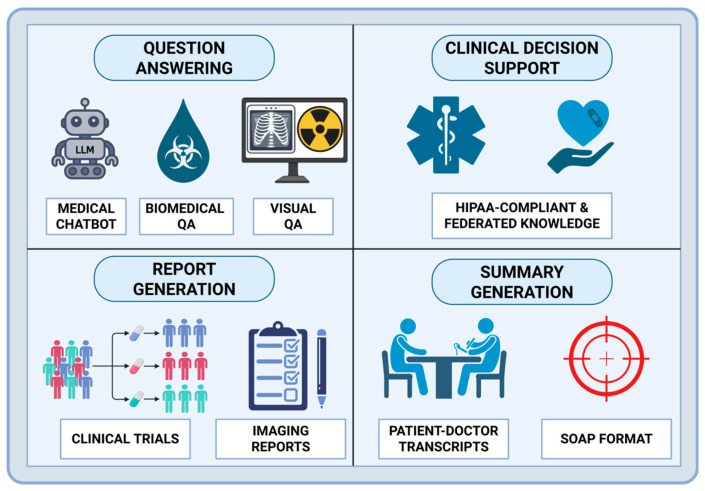
Healthcare applications of Hybrid RAG + FT architectures. Applications include QA (medical chatbots, biomedical QA, and visual QA), clinical decision support leveraging HIPAA-compliant and federated knowledge sources, automated report generation for clinical trials and imaging studies, and clinical summary generation from patient–doctor transcripts using structured formats such as SOAP notes. Created in BioRender. Collaco, B. (2025) https://BioRender.com/ecvmjvt.

Across the included studies, a dominant motivation for adopting hybrid FT + RAG is reducing hallucinations and improving factual consistency, particularly for tasks where errors can plausibly affect patient safety and downstream decision-making. Empirical clinical evidence highlights how treatment decisions and practice patterns evolve over time and vary across institutions, even within the same clinical context, as demonstrated by longitudinal analyses of neonatal antibiotic use in tertiary care settings [42]. In this subclass of systems, retrieval is generally positioned as a mechanism for accessing up-to-date or verifiable medical knowledge, while FT (often domain- or task-adaptive) is used to strengthen the model’s ability to interpret and apply that information within the target clinical workflow. Within the limits of the available evidence, this complementarity is reported to support performance improvements in applications such as medical QA, clinical summarization, and automated report generation [8,18,19,20,21,22,23], where grounding failures may directly impact patient safety (Figure 3). Taken together, these findings indicate that neither static FT nor retrieval alone may be sufficient to consistently meet clinical accuracy requirements at scale [43]. However, solid conclusions about superiority over FT-only or RAG-only systems remain limited given the small number of studies and heterogeneous evaluation designs.

A major constraint identified across studies is the difficulty of evaluating hybrid systems in a way that cleanly attributes gains and failures to the retriever versus the generator. Several authors emphasize that hybrid evaluation should adopt a two-layer perspective, assessing retriever behavior (e.g., recall/precision and evidence quality) alongside generator behavior (e.g., factual accuracy, hallucination rate, and faithfulness), because improvements in one module can mask failures in the other [41]. Consistent with this, the included literature shows a strong reliance on automated metrics, while expert or human judgments are less frequent despite their importance for clinical relevance, completeness, and safety. This evaluation imbalance contributes to the current difficulty of conducting standardized, head-to-head comparisons across integrated FT + RAG systems and limits the strength of cross-study inference.

Within the FT component, the included studies show a pronounced tendency toward PEFT techniques, such as LoRA and QLoRA, rather than full-parameter FT. This convergence reflects shared practical constraints across healthcare settings, including limited computational resources, the need for rapid iteration, and institutional barriers to large-scale model retraining. From an application standpoint, PEFT enables healthcare organizations to adapt general-purpose LLMs to local clinical tasks, such as documentation, coding, or specialty-specific decision support, while maintaining feasibility for deployment and maintenance [44,45]. Cross-domain PEFT synthesis further supports this interpretation, reporting that PEFT methods often achieve results on par with full FT and sometimes better, while also improving training and/or memory efficiency; LoRA-based methods, particularly LoRA and QLoRA, are highlighted as widely adopted and effective across multiple generative tasks. Although these findings originate outside healthcare, they help explain why PEFT has emerged as the default FT substrate for hybrid clinical systems operating under tight computational and governance constraints [44,45,46]. However, the limited evidence base for integrated FT + RAG systems means that direct clinical comparisons between full FT and PEFT within hybrid pipelines remain an important open research need, especially in healthcare AI.

In contrast to the relative standardization of FT strategy, RAG implementations in the included studies exhibit substantial architectural variability, spanning dense vector retrieval, hybrid retrieval, hierarchical retrieval, multimodal retrieval, and federated retrieval. This diversity suggests that, in explicitly integrated FT + RAG systems, retrieval is being actively tailored to clinical context rather than treated as a uniform or auxiliary component. For example, dense RAG pipelines are more commonly applied to medical QA [8,18,21,23], whereas hierarchical and multimodal retrieval architectures are preferentially used for more complex tasks such as clinical trial reporting [20], radiology interpretation, and multimodal reasoning [19], where relevant information is distributed across unstructured text, structured records, and medical images. This aligns with the practical reality that clinical information ecosystems are fragmented and multimodal, and it supports the view that retrieval depth and structure should be considered a controllable policy rather than a fixed configuration [47].

Finally, several included studies extend integrated FT + RAG into federated and ontology-driven settings, reinforcing the central role of privacy preservation, interpretability, and institutional governance in healthcare AI [19]. Federated FT and federated retrieval are positioned as mechanisms to leverage cross-institutional knowledge without exposing raw patient data, addressing regulatory and ethical constraints that are often decisive in real deployments. These directions highlight a promising pathway for multi-site clinical decision support and collaborative diagnostics; however, they also raise open questions about evaluation standardization, security threat models, and the operational costs of maintaining retrieval resources and fine-tuned adapters over time.

Overall, this review maps an emerging set of explicitly integrated FT + RAG healthcare systems and identifies key gaps that currently limit generalization: (I) a small empirical evidence base, (II) inconsistent and often incomplete evaluation across retriever and generator components, and (III) limited comparative testing against well-matched FT-only and RAG-only baselines under clinically meaningful conditions. Addressing these gaps will be essential to determine when hybridization offers robust advantages and to define best practices for safe, maintainable deployment.

### 4.3. Strengths and Limitations

A key strength of this scoping review lies in its focused and methodologically explicit definition of hybrid FT + RAG frameworks in healthcare AI. By restricting inclusion to systems that combine model-level parameter adaptation with retrieval-based knowledge grounding at inference time, this review isolates a conceptually distinct and increasingly relevant subclass of LLM-based healthcare systems. This deliberate abstraction enables meaningful cross-study comparison despite substantial heterogeneity in underlying architectures, datasets, and clinical tasks. Additionally, the application-oriented synthesis of our findings rather helps finding emerging trends and identifying evidence gaps in healthcare AI.

Nonetheless, there are several limitations that should be considered when interpreting its findings. First, the small number of included studies limits the ability to solid conclusions of the statements provided. However, this constraint reflects the current state of the literature, as fully integrated FT + RAG frameworks remain relatively uncommon in empirically validated healthcare applications, rather than a limitation of the search strategy itself.

Second, the intentional exclusion of loosely coupled, partially hybrid, and individual approaches, such as RAG-only and FT-only pipelines, may omit adjacent work that explores related concepts. This choice was made to preserve methodological clarity and comparability, but it necessarily narrows the scope of inference to explicitly integrated hybrid architectures.

Finally, substantial heterogeneity in evaluation metrics, datasets, and reporting practices across included studies limits direct performance comparison and trend quantification. Most studies rely on task-specific benchmarks or expert preference assessments, and few report standardized safety, robustness, or longitudinal outcomes. As a result, conclusions regarding relative effectiveness should be interpreted qualitatively rather than as definitive performance rankings.

### 4.4. Future Directions

The present literature suggests that future progress will depend less on incremental performance gains and more on improved orchestration of adaptation strategies. Promising directions include RAG-aware FT, adaptive retrieval policies, clinician-in-the-loop alignment, and standardized evaluation frameworks that capture safety, interpretability, and workflow impact [10,33]. This emphasis reflects a broader maturation of healthcare AI, with hybrid frameworks moving from proof-of-concept models toward systems that are deployable, governable, and clinically credible. As this methodological space matures, future reviews may achieve broader coverage by incorporating newly published empirical studies and, where appropriate, expanding inclusion criteria to encompass more loosely coupled hybrid designs. However, the added architectural complexity of hybrid RAG + FT systems necessitates further research into uncertainty quantification, clinician-facing rationale generation, and automated provenance tracking [48]. Integration with clinical workflows and digital infrastructure will also be critical for real-world adoption.

Future studies should evaluate model performance under continuous learning or versioned updates, particularly within emerging regulatory frameworks such as the FDA’s Predetermined Change Control Plan (PCCP) and the EU AI Act, to better understand long-term stability, monitoring requirements, and operational safeguards for safe clinical deployment [49,50]. Alignment with these regulatory frameworks may also facilitate the future integration of agentic AI architectures into hybrid RAG + FT systems. Agentic frameworks extend conventional LLM pipelines by enabling models to autonomously plan, decompose tasks, invoke tools, and iteratively reason over intermediate outputs. In healthcare settings, such capabilities could allow hybrid systems to dynamically orchestrate retrieval, fine-tuned reasoning, validation, and escalation to human oversight based on task complexity or uncertainty [51]. For example, an agentic system could determine when additional evidence retrieval is required, select appropriate clinical knowledge sources, request clinician input for ambiguous cases, or defer actions when confidence thresholds are not met. When combined with RAG and FT, agentic AI has the potential to transform static decision-support tools into adaptive, workflow-aware assistants capable of supporting longitudinal clinical processes [17,52].

## 5. Conclusions

This scoping review maps the current landscape of explicitly integrated FT + RAG frameworks in healthcare AI, highlighting an emerging but still limited body of empirical evidence. Across the included studies, hybrid architectures appear to offer a promising balance between domain-specific reasoning and dynamic knowledge grounding, addressing key clinical challenges such as hallucination reduction, factual reliability, and adaptability to evolving medical practice. At the same time, the small number of empirically validated systems, substantial heterogeneity in evaluation practices, and limited real-world deployment underscore that hybrid FT + RAG integration remains an early-stage methodological direction rather than a mature standard. By synthesizing cross-study trends, identifying architectural and evaluation patterns, and delineating critical evidence gaps, this review clarifies both the current potential and the constraints of hybrid FT + RAG approaches in healthcare. As the field matures, continued empirical validation, standardized evaluation frameworks, and closer alignment with clinical workflows and regulatory requirements will be essential for translating these hybrid systems into safe, effective, and clinically credible AI tools.

## Figures and Tables

**Table 1:** Baseline characteristics of included studies employing hybrid FT and RAG frameworks in healthcare AI.

Author, Year	Country	Hybrid Framework				
		Name ^a^	Base Model	FT Strategy	RAG Strategy	Main Clinical Task
Bora and Cuayáhuitl, 2024 [18]	UK	RAG-based medical chatbot	LLaMA-2-7B, Mistral-7B, Fran-T5-Large	PEFT (LoRA)	Dense RAG	Medical chatbot QA
Garcia et al., 2025 [19]	USA	DF-RAG	LLaMA, DeepSeek, Qwen ^b^	Federated PEFT (FlexLoRA)	FKG-based RAG	Clinical decision support (theoretical)
Kuo et al., 2025 [20]	Taiwan	Multimodal RAG–LLM	LLaMA-3 8B-Instruct	PEFT (LoRA/QLoRA) + RL alignment	Multimodal Hierarchical RAG	Automated clinical trial report generation
Gao et al., 2024 [21]	China	CPS	LlaMA-2-7B	PEFT (LoRA)	Hierarchical Hybrid RAG (Sparse + Dense)	Biomedical QA
Neupane et al., 2024 [22]	USA	CLINICSUM	LLaMA-3-8B, Gemma-2-9B, Mistral-7B, Mistral-Nemo-12B	PEFT (LoRA)	Hybrid RAG (Sparse + Dense)	SOAP clinical summary generation
Pingua et al., 2025 [8]	USA and India	Hybrid FT + RAG approach	Meta-LLaMA-3.1-8B; Phi-3.5-Mini-Instruct; Gemma-2-9B; Mistral-7B-Instruct; Qwen2.5-7B	PEFT (LoRA/QLoRA)	Dense RAG	Medical QA
Xia et al., 2024 [23]	USA	MMed-RAG	LLaVA-Med-1.5 (7B)	RAG-aware PEFT (LoRA) + DPO	Multimodal RAG with domain-aware retrieval	Medical VQA and medical report generation across radiology, ophthalmology, and pathology

^a^ System’s name or how it is presented. ^b^ Did not specify which one. DF-RAG = Dual Federated Retrieval-Augmented Generation; FKG = Federated Knowledge Graph; CPS = Corpus PEFT Searching; MMed-RAG = Multimodal Medical RAG; LLaMA = Large Language Model Meta AI; PEFT = Parameter Efficient Fine-Tuning; LoRA = Low-Rank Adaptation; QLoRA = Quantized Low Rank Adaptation; DPO = Direct Preference Optimization; SOAP = Subjective, Objective, Assessment, and Plan; QA = Question Answering; VQA = Visual Question Answering.

**Table 2:** Qualitative and quantitative comparison of hybrid frameworks evaluated across healthcare main tasks, models, and datasets.

Author, Year	Baseline Model	Key Metrics	Quantitative Performance (Dataset)	Main Qualitative Findings
**Bora and Cuayáhuitl, 2024** [18]	Flan-T5-Large	MCQ type A: Exact Match (%); MCQ Type B: MRR/LaCC@1	33% (Meadow-MedQA)/26% (MedMCQA) 0.44/21.42% (Meadow-MedQA) and 0.43/18.6% (MedMCQA)	Mistral-7B consistently outperformed the other models across various tasks and configurations. It demonstrated superior accuracy, relevance, and overall performance. Flan-T5- Large showed lower accuracy and performance across tasks and the fastest processing. Llama-2-7B offered a balanced performance.
	LLaMA-2-7B		51% (Meadow-MedQA)/55% (MedMCQA) 0.44/22.8% (Meadow-MedQA) and 0.47/26.2% (MedMCQA)	
	Mistral-7B		54% (Meadow-MedQA)/57% (MedMCQA) 0.51/0.7% (Meadow-MedQA) and 0.53/32.8% (MedMCQA)	
**Garcia et al., 2025** [19]	Seed LLM ^a^	Privacy (1–5) Collaboration (1–5) Accuracy (1–5) Explainability (1–5) Regulation (1–5) Scalability (1–5)	Total score: 28/30	Improved accuracy, privacy, interpretability in theoretical evaluation. Highest score compared with other non-hybrid approaches.
**Kuo et al., 2025** [20]	LLaMA-3 8B-Instruct	RG: ROUGE-L/BERTScore/Med-Concept F1/FactCC-Med/CQI;	43.1/0.904/0.791/6.2/78.3	Improved factual grounding with reduced hallucinations, high clinician acceptance, strong workflow integration, enhanced trust via traceable evidence and expert review. Improved report quality with 75% generation time reduction
**Gao et al., 2024** [21]	LlaMA-2-7B	Biomedical QA: ROUGE-2/ROUGE-SU4	0.558/0.573 (BioASQ Task 11b)	Improved QA performance; FT + RAG outperformed baselines
**Neupane et al, 2024** [22]	LLaMA-3-8B	SOAP: ROUGE-1/ROUGE-2/ROUGE-L/BERTScore-F1	0.70/0.48/0.55/0.84	Outperformed GPT models; higher factual accuracy and 61% SME preference
	Gemma-2-9B		0.67/0.43/0.49/0.82	
	Mistral-7B		0.68/0.45/0.49/0.79	
	Mistral-Nemo-12B		0.68/0.45/0.51/0.78	
**Pingua et al., 2025** [8]	LLaMA-3.1-8B	QA: BLEU/GLEU/ROUGE-1/ROUGE-2/ROUGE-L/METEOR/BERTScore-F1/SB_CS/NASS	0.153/0.236/0.423/0.299/0.413/0.268/0.891/0.819/0.908	FT + RAG significantly outperforms FT across BLEU, ROUGE, BERTScore, and NASS; strongest gains for LLaMA-3.1-8B and Phi-3.5-Mini (*p* < 0.05). RAG + FT improves factual grounding, semantic coherence, and negation handling; benefits are model-dependent and sometimes comparable to RAG alone
	Phi-3.5-Mini-Instruct		0.136/0.217/0.391/0.250/0.380/0.258/0.876/0.815/0.881	
	Gemma-2-9B		0.104/0.181/0.351/0.193/0.338/0.240/0.871/0.791/0.841	
	Mistral-7B-Instruct		0.089/0.172/0.346/0.205/0.331/0.207/0.875/0.796/0.857	
	Qwen-2.5-7B		0.042/0.130/0.290/0.160/0.872/0.787/0.859	
**Xia et al., 2024** [23]	LLaVA-Med-1.5 (7B)	VQA: Accuracy/F1/AUROC RG: BLEU/ROUGE-L/METEOR	**R VQA:** 89.54/80.72/87.13 (IU-Xray); 83.57/88.49/85.08 (MIMIC-CXR) **O VQA:** 87.94/92.78/80.81 (Harvard-FairVLMed) **P VQA:** 72.95/76.35/72.25 (Quilt-1M); 64.54/73.09/61.42 (PMC-OA); **R RG:** 31.38/25.59/32.43 (IU-Xray); 23.25/12.34/20.47 (MIMIC-CXR) **O RG:** 24.82/16.59/19.85 (Harvard-FairVLMed)	Improved factual accuracy by 18.5% (VQA) and 69.1% (RG) over baseline Med-LVLM; outperformed decoding-based and prior RAG methods; reduced hallucinations and improved cross-modality alignment

^a^ Did not specify which one. MCQ = multiple-choice questions; SME = Subject Matter Experts; MRR = Mean Reciprocal Rank; SB_CS = SBERT Cosine Similarity; NASS = Negation-Aware Semantic Similarity; CQI = Composite Quality Index; SOAP = Subjective, Objective, Assessment, and Plan; QA = Question Answering; VQA = Visual Question Answering. RG = Report generation; R = Radiology; O = Ophthalmology; P = Pathology.

## Data Availability

The data supporting the findings of this review are available within the article and its Supplementary Materials. Extracted information from the included studies is summarized in the main text and tables.

## Supplementary Materials

The following supporting information can be downloaded at: https://www.mdpi.com/article/10.3390/bioengineering13020225/s1, Supplementary File S1: Complete search strategy across databases.

## References

[1] Haider S.A., Prabha S., Gomez-Cabello C.A., Borna S., Genovese A., Trabilsy M., Collaco B.G., Wood N.G., Bagaria S., Tao C. (2025). Synthetic Patient–Physician Conversations Simulated by Large Language Models: A Multi-Dimensional Evaluation. Sensors.

[2] Zhang K., Meng X., Yan X., Ji J., Liu J., Xu H., Zhang H., Liu D., Wang J., Wang X. (2025). Revolutionizing Health Care: The Transformative Impact of Large Language Models in Medicine. J. Med. Internet Res..

[3] McCoy L.G., Swamy R., Sagar N., Wang M., Bacchi S., Fong J.M.N., Tan N.C., Tan K., Buckley T.A., Brodeur P. (2025). Assessment of large language models in clinical reasoning: A novel benchmarking study. NEJM AI.

[4] Asgari E., Montaña-Brown N., Dubois M., Khalil S., Balloch J., Yeung J.A., Pimenta D. (2025). A framework to assess clinical safety and hallucination rates of LLMs for medical text summarisation. npj Digit. Med..

[5] Agarwal V., Jin Y., Chandra M., De Choudhury M., Kumar S., Sastry N. (2024). Medhalu: Hallucinations in responses to healthcare queries by large language models. arXiv.

[6] Wang Z., Wang H., Danek B., Li Y., Mack C., Arbuckle L., Biswal D., Poon H., Wang Y., Rajpurkar P. (2025). A perspective for adapting generalist ai to specialized medical ai applications and their challenges. npj Digit. Med..

[7] Ding H., Fang Y., Zhu R., Jiang X., Zhang J., Xu Y., Liao W., Chu X., Zhao J., Wang Y. (2025). 3DS: Medical Domain Adaptation of LLMs via Decomposed Difficulty-based Data Selection. Proceedings of the 2025 Conference on Empirical Methods in Natural Language Processing.

[8] Pingua B., Sahoo A., Kandpal M., Murmu D., Rautaray J., Barik R.K., Saikia M.J. (2025). Medical LLMs: Fine-Tuning vs. Retrieval-Augmented Generation. Bioengineering.

[9] Ng K.K.Y., Matsuba I., Zhang P.C. (2025). RAG in health care: A novel framework for improving communication and decision-making by addressing LLM limitations. NEJM AI.

[10] Amugongo L.M., Mascheroni P., Brooks S., Doering S., Seidel J. (2025). Retrieval augmented generation for large language models in healthcare: A systematic review. PLoS Digit. Health.

[11] Haider S.A., Prabha S., Gomez Cabello C.A., Genovese A., Collaco B., Wood N., London J., Bagaria S., Tao C., Forte A.J. (2025). The Development and Evaluation of a Retrieval-Augmented Generation Large Language Model Virtual Assistant for Postoperative Instructions. Bioengineering.

[12] Rangan K., Yin Y. (2024). A fine-tuning enhanced RAG system with quantized influence measure as AI judge. Sci. Rep..

[13] Soudani H., Kanoulas E., Hasibi F. (2024). Fine tuning vs. retrieval augmented generation for less popular knowledge. Proceedings of the 2024 Annual International ACM SIGIR Conference on Research and Development in Information Retrieval in the Asia Pacific Region.

[14] Aromataris E., Munn Z. (2020). Chapter 11: Scoping reviews. JBI Reviewer’s Manual.

[15] Page M.J., McKenzie J.E., Bossuyt P.M., Boutron I., Hoffmann T.C., Mulrow C.D., Shamseer L., Tetzlaff J.M., Akl E.A., Brennan S.E. (2021). The PRISMA 2020 statement: An updated guideline for reporting systematic reviews. BMJ.

[16] Light M. (2010). EndNote 1-2-3 Easy! Reference Management for the Professional, Second Edition, A. Agrawal, 2009, Springer, New York, USA, Price: €44.95, Soft Cover, 294 pages, ISBN: 978-0-387-95900-9, Website: www.springer.com. S. Afr. J. Bot..

[17] Collaco B.G., Haider S.A., Prabha S., Gomez-Cabello C.A., Genovese A., Wood N.G., Bagaria S.P., Gopala N., Tao C., Forte A.J. (2025). The Role of Agentic Artificial Intelligence in Healthcare: A Systematic Review. Preprint.

[18] Bora A., Cuayáhuitl H. (2024). Systematic Analysis of Retrieval-Augmented Generation-Based LLMs for Medical Chatbot Applications. Mach. Learn. Knowl. Extr..

[19] Garcia J., Gong J., Zajac M., Hahn A. (2025). DF-RAG: A Dual Federated Retrieval-Augmented Generation Framework for Collaborative Medical AI. Proceedings of the ACM/IEEE International Conference on Connected Health: Applications, Systems and Engineering Technologies.

[20] Kuo S.-M., Tai S.-K., Lin H.-Y., Chen R.-C. (2025). Automated Clinical Trial Data Analysis and Report Generation by Integrating Retrieval-Augmented Generation (RAG) and Large Language Model (LLM) Technologies. AI.

[21] Gao Y., Zong L., Li Y. (2024). Enhancing biomedical question answering with parameter-efficient fine-tuning and hierarchical retrieval augmented generation. CLEF Working Notes.

[22] Neupane S., Tripathi H., Mitra S., Bozorgzad S., Mittal S., Rahimi S., Amirlatifi A. (2024). ClinicSum: Utilizing Language Models for Generating Clinical Summaries from Patient-Doctor Conversations. Proc. IEEE Int. Conf. Big Data.

[23] Xia P., Zhu K., Li H., Wang T., Shi W., Wang S., Zhang L., Zou J., Yao H. (2024). Mmed-rag: Versatile multimodal rag system for medical vision language models. arXiv.

[24] Hou Z., Liu H., Bian J., He X., Zhuang Y. (2025). Enhancing medical coding efficiency through domain-specific fine-tuned large language models. npj Health Syst..

[25] Nunes M., Bone J., Ferreira J.C., Elvas L.B. (2024). Health Care Language Models and Their Fine-Tuning for Information Extraction: Scoping Review. JMIR Med. Inform..

[26] Maity S., Saikia M.J. (2025). Large Language Models in Healthcare and Medical Applications: A Review. Bioengineering.

[27] Dorfner F.J., Dada A., Busch F., Makowski M.R., Han T., Truhn D., Kleesiek J., Sushil M., Adams L.C., Bressem K.K. (2025). Evaluating the effectiveness of biomedical fine-tuning for large language models on clinical tasks. J. Am. Med. Inform. Assoc..

[28] Gargari O.K., Habibi G. (2025). Enhancing medical AI with retrieval-augmented generation: A mini narrative review. Digit. Health.

[29] Eastwood B. How Does Retrieval-Augmented Generation (RAG) Support Healthcare AI Initiatives?. https://healthtechmagazine.net/article/2025/01/retrieval-augmented-generation-support-healthcare-ai-perfcon.

[30] Yang R., Ning Y., Keppo E., Liu M., Hong C., Bitterman D.S., Ong J.C.L., Ting D.S.W., Liu N. (2025). Retrieval-augmented generation for generative artificial intelligence in health care. npj Health Syst..

[31] Genovese A., Prabha S., Borna S., Gomez-Cabello C.A., Haider S.A., Trabilsy M., Ho O.A., Forte A.J. (2025). 34. Assessing the Effectiveness of RAG-Based AI Models in Answering Postoperative Rhinoplasty Queries: Limitations and Future Directions. Plast. Reconstr. Surg.–Glob. Open.

[32] Ke Y.H., Jin L., Elangovan K., Abdullah H.R., Liu N., Sia A.T.H., Soh C.R., Tung J.Y.M., Ong J.C.L., Kuo C.-F. (2025). Retrieval augmented generation for 10 large language models and its generalizability in assessing medical fitness. npj Digit. Med..

[33] Liu S., McCoy A.B., Wright A. (2025). Improving large language model applications in biomedicine with retrieval-augmented generation: A systematic review, meta-analysis, and clinical development guidelines. J. Am. Med. Inform. Assoc..

[34] Lakatos R., Pollner P., Hajdu A., Joo T. (2025). Investigating the performance of retrieval-augmented generation and domain-specific fine-tuning for the development of AI-driven knowledge-based systems. Mach. Learn. Knowl. Extr..

[35] Shi Y., Xu S., Yang T., Liu Z., Liu T., Li X., Liu N. (2024). MKRAG: Medical Knowledge Retrieval Augmented Generation for Medical Question Answering. AMIA Annu. Symp. Proc..

[36] Xiong G., Jin Q., Wang X., Zhang M., Lu Z., Zhang A. (2024). Improving retrieval-augmented generation in medicine with iterative follow-up questions. Biocomputing 2025: Proceedings of the Pacific Symposium.

[37] Lu S., Cosgun E. (2025). Boosting GPT models for genomics analysis: Generating trusted genetic variant annotations and interpretations through RAG and Fine-tuning. Bioinform. Adv..

[38] Zhang T., Patil S.G., Jain N., Shen S., Zaharia M., Stoica I., Gonzalez J.E. (2024). Raft: Adapting language model to domain specific rag. arXiv.

[39] Lopez I., Swaminathan A., Vedula K., Narayanan S., Nateghi Haredasht F., Ma S.P., Liang A.S., Tate S., Maddali M., Gallo R.J. (2025). Clinical entity augmented retrieval for clinical information extraction. npj Digit. Med..

[40] Elkin P.L., Mehta G., LeHouillier F., Koppel R., Elkin A.N., Nebeker J., Brown S.H. (2025). Retrieval Augmented Generation: What Works and Lessons Learned. Stud. Health Technol. Inform..

[41] Brown A., Roman M., Devereux B. (2025). A systematic literature review of retrieval-augmented generation: Techniques, metrics, and challenges. Big Data Cogn. Comput..

[42] Dorobanțu F.R., Hodoșan V., Tîrb A.M., Zaha D.C., Galușca D., Pop N.O., Dorobanțu C.D. (2022). Pattern of newborn antibiotic use in a tertiary level maternity for five years. Pharmacophore.

[43] Das S., Ge Y., Guo Y., Rajwal S., Hairston J., Powell J., Walker D., Peddireddy S., Lakamana S., Bozkurt S. (2024). Two-layer retrieval augmented generation framework for low-resource medical question-answering: Proof of concept using Reddit data. arXiv.

[44] Hu E.J., Shen Y., Wallis P., Allen-Zhu Z., Li Y., Wang S., Wang L., Chen W. (2022). Lora: Low-rank adaptation of large language models. ICLR.

[45] Dettmers T., Pagnoni A., Holtzman A., Zettlemoyer L. (2023). Qlora: Efficient finetuning of quantized llms. Adv. Neural Inf. Process. Syst..

[46] Afrin S., Haque M.Z., Mastropaolo A. (2025). A systematic literature review of parameter-efficient fine-tuning for large code models. arXiv.

[47] Zhao S., Yang Y., Wang Z., He Z., Qiu L.K., Qiu L. (2024). Retrieval augmented generation (rag) and beyond: A comprehensive survey on how to make your llms use external data more wisely. arXiv.

[48] Atf Z., Safavi-Naini S.A.A., Lewis P.R., Mahjoubfar A., Naderi N., Savage T.R., Soroush A. (2025). The challenge of uncertainty quantification of large language models in medicine. arXiv.

[49] González C., Fuchs M., Santos D.P.d., Matthies P., Trenz M., Grüning M., Chaudhari A., Larson D.B., Othman A., Kim M. (2024). Regulating radiology AI medical devices that evolve in their lifecycle. arXiv.

[50] Farah L., Borget I., Martelli N. (2023). International Market Access Strategies for Artificial Intelligence–Based Medical Devices: Can We Standardize the Process to Faster Patient Access?. Mayo Clin. Proc. Digit. Health.

[51] Yuan H. (2025). Agentic large language models for healthcare: Current progress and future opportunities. Med. Adv..

[52] Vatsal S., Dubey H., Singh A. (2025). Agentic AI in Healthcare & Medicine: A Seven-Dimensional Taxonomy for Empirical Evaluation of LLM-based Agents. arXiv.

