# Supplementary material for: Integrating Fine-Tuning and Retrieval-Augmented Generation for Healthcare AI Systems: A Scoping Review
Source: Bioengineering (Basel). 2026 Feb 14;13(2):225. doi: 10.3390/bioengineering13020225 (PMC12938813; doi:10.3390/bioengineering13020225)
Supplement: Supplementary file 1 [file bioengineering-13-00225-s001.zip › bioengineering-4128320-Supplementary File S1.pdf]

Pubmed, IEEE Xplore, Google Scholar > ("fine-tuning" OR "fine tuning" OR "parameter-efficient fine-tuning" OR PEFT OR LoRA OR QLoRA) and ("retrieval-augmented generation" OR "retrieval augmented generation" OR RAG) AND (healthcare OR patient OR medicine OR clinical OR biomedical OR medical OR "electronic health record" OR EHR)

Embase > ('fine-tuning' OR 'fine tuning' OR 'parameter-efficient fine-tuning' OR 'peft' OR 'lora' OR 'qlora') AND ('retrieval-augmented generation' OR 'retrieval augmented generation' OR 'rag') AND ('healthcare' OR 'patient' OR 'medicine' OR 'clinical' OR 'biomedical' OR 'medical' OR 'electronic health record' OR 'ehr')
